# SARS-CoV-2-Specific CD8^+^ T-Cells in Blood but Not in the Lungs of Vaccinated K18-hACE2 Mice after Infection

**DOI:** 10.3390/vaccines11091433

**Published:** 2023-08-30

**Authors:** Flavia Ferrantelli, Francesco Manfredi, Chiara Chiozzini, Patrizia Leone, Katherina Pugliese, Massimo Spada, Antonio Di Virgilio, Andrea Giovannelli, Mauro Valeri, Andrea Cara, Zuleika Michelini, Mauro Andreotti, Maurizio Federico

**Affiliations:** 1 National Center for Global Health, Istituto Superiore di Sanità, Viale Regina Elena 299, 00161 Rome, Italy; flavia.ferrantelli@iss.it (F.F.); francesco.manfredi@iss.it (F.M.); chiara.chiozzini@iss.it (C.C.); patrizia.leone@iss.it (P.L.); katherina.pugliese@iss.it (K.P.); andrea.cara@iss.it (A.C.); zuleika.michelini@iss.it (Z.M.); mauro.andreotti@iss.it (M.A.); 2 National Center for Animal Experimentation and Welfare, Istituto Superiore di Sanità, Viale Regina Elena 299, 00161 Rome, Italy; massimo.spada@iss.it (M.S.); antonio.divirgilio@iss.it (A.D.V.); andrea.giovannelli@iss.it (A.G.); mauro.valeri@iss.it (M.V.)

**Keywords:** SARS-CoV-2, CD8^+^ T-cell immunity, vaccines, K18 transgenic mice

## Abstract

Severe acute respiratory syndrome coronavirus (SARS-CoV)-2 enters the host by infecting nasal ciliated cells. Then, the virus can spread towards the oropharyngeal cavity and the pulmonary tissues. The antiviral adaptive immunity is promptly induced in response to the virus’s detection, with virus-specific T-lymphocytes appearing before antiviral antibodies. Both the breadth and potency of antiviral CD8^+^ T-cell immunity have a key role in containing viral spread and disease severity. Current anti-SARS-CoV-2 vaccines do not impede the virus’s replication in the upper respiratory tract, and there is consensus on the fact that the best potency of the antiviral immune response in both blood and the upper respiratory tract can be reached upon infection in vaccinees (i.e., breakthrough infection). However, whether the antiviral CD8^+^ T-cells developing in response to the breakthrough infection in the upper respiratory tract diffuse to the lungs is also still largely unknown. To fill the gap, we checked the CD8^+^ T-cell immunity elicited after infection of K18-hACE2 transgenic mice both at 3 weeks and 3 months after anti-spike vaccination. Virus-specific CD8^+^ T-cell immunity was monitored in both blood and the lungs before and after infection. By investigating the de novo generation of the CD8^+^ T-cells specific for SARS-CoV-2 viral proteins, we found that both membrane (M) and/or nucleocapsid (N)-specific CD8^+^ T-cells were induced at comparable levels in the blood of both unvaccinated and vaccinated mice. Conversely, N-specific CD8^+^ T-cells were readily found in the lungs of the control mice but were either rare or absent in those of vaccinated mice. These results support the idea that the hybrid cell immunity developing after asymptomatic/mild breakthrough infection strengthens the antiviral cell immunity in the lungs only marginally, implying that the direct exposition of viral antigens is required for the induction of an efficient antiviral cell immunity in the lungs.

## 1. Introduction

Studies on both animals and humans demonstrated that CD8^+^ T-cell immunity plays a key role in recovery from severe acute respiratory syndrome coronavirus (SARS-CoV)-2 disease [1]. Its efficacy is largely unaffected by the amino acid substitutions occurring in emerging viral variants [2], and the infection with low pathogenic coronaviruses can generate an effective anti-SARS-CoV-2 CD8^+^ T-cell immunity due to the cross-reactivity of virus-specific CD8^+^ T-cells [3].

For instance, the role of antiviral CD8^+^ T-cell immunity in the control of the SARS-CoV-2 infection was highlighted by a study in rhesus macaques, which demonstrated that the depletion of CD8^+^ T-cells after a first-virus challenge abolished the protective effect of natural immunity against a virus re-challenge carried out after the waning of neutralizing antibodies [4]. In humans, the presence of cytotoxic CD8^+^ T-cells specific for a SARS-CoV-2 N immunodominant epitope (i.e., the HLA-B07-restricted N_105–113_) is associated with the development of a mild disease [5]. Moreover, oncologic patients with significant impairment of B cells but preserved CD8^+^ T-cell counts showed lower viral loads and reduced mortality after SARS-CoV-2 infection compared to what was observed in homologous patients with low CD8^+^ T-cell counts. In addition, the depletion of B cells in patients with hematologic cancers was not associated with increased COVID-19-related mortality [6].

Persons completing the anti-SARS-CoV-2 vaccination cycles remain susceptible to the infection, showing peaks of viral load in the upper respiratory tract similar to those detected in unvaccinated persons [7,8]. Several lines of evidence indicated that the vaccine breakthrough infection (BTI) generates a sharp improvement in the antiviral immune response in both blood and the upper respiratory tract with respect to that present in uninfected vaccines [9]. It would be of interest to evaluate whether a BTI can strengthen antiviral CD8^+^ T-cell immunity in the lungs as a possible consequence of the increased cell immunity in the blood. The issue is of relevance, considering that current anti-SARS-CoV-2 vaccines induce quite poor antiviral cell immunity in the lower respiratory tract [10]. We attempted to shed light on this issue through the analysis of the CD8^+^ T-cell immune response after infection of vaccinated K18-hACE2 mice. SARS-CoV-2 replicates in these animals by virtue of the expression in epithelial cells of the human receptor of the SARS-CoV-2 virus, i.e., the human angiotensin-converting enzyme (hACE)-2, under the control of the cytokeratin-18 promoter [11]. The intranasal infection with SARS-CoV-2 leads to a sustained viral replication in both nasal turbinate and the lungs as early as 24 h post-infection [12].

Here, we monitored the induction of antiviral CD8^+^ T-cell immunity in both the blood and lungs of vaccinated K18-hACE2 mice after infection.

## 2. Materials and Methods

### 2.1. Animals and Authorizations

Six-week-old female C57 Bl/6 K18-hACE2 transgenic mice were purchased from Charles River (Calco, Italy) and hosted at the Central and BSL3 Animal Facilities of the Istituto Superiore di Sanità. The study was conducted according to the guidelines of the Declaration of Helsinki and approved by the Italian Ministry of Health, authorization 591/2021, released on 30 July 2021. The authors complied with the ARRIVE guidelines, including the use of control mice shared with already reported heterologous experiments [13]. Before the first procedure, Datamars (Lugano, Switzerland) microchips were inserted subcutaneously in the dorsal midline between the shoulder blades.

### 2.2. Anti-Spike Vaccines

The Ph-CMV DNA vector expressing a full-length, codon-optimized wild-type SARS-CoV-2 spike protein open reading frame (ORF) (Wuhan-Hu-1, GenBank: NC_045512.2) [14] was used as a DNA vaccine. Mice were also immunized with the mRNA-based anti-spike Moderna (Cambridge, MA, USA) Spikevax vaccine, lot # 000030A, 0.2 mg/mL.

### 2.3. Mice Immunization

Isoflurane-anesthetized mice were inoculated i.m. with 10 μg of DNA in 30 μL of sterile 0.9% saline solution. The DNA injection was immediately followed by electroporation at the site of inoculation with an Agilpulse BTX (Holliston, MA, USA) device, using a four-needle electrode array (4 mm gap, 5 mm needle length), and applying the following parameters: 1 pulse of 450 V for 50 μs with a 0.2 ms interval; 1 pulse of 450 V for 50 μs with a 50 ms interval; 8 pulses of 110 V for 10 ms with 20 ms intervals. Mice in the Spikevax-immunization group were first mock-inoculated with 30 μL of sterile, 0.9% saline solution followed by electroporation, as described above, and then vaccinated by i.m. injection into a different site (out of the electroporated area) with 30 µL of Spikevax containing 0.5 μg of RNA. The mice were immunized into both quadriceps twice, 2 weeks apart. The mice were sacrificed by cervical dislocation.

### 2.4. Anti-Spike ELISA

The SARS-CoV-2 recombinant S1 protein (Sigma, St. Louis, MO, USA, AGX 818) was used for coating 96 well plates (Greiner bio-one, Frickenhausen, Germany) with 0.1 µg/well of protein overnight at 4 °C. The plates were washed and blocked with 1% BSA (Sigma Chemicals) in PBS. Duplicate wells of 2-fold serial dilutions of plasma from each mouse were incubated for 2 h at room temperature. After washing, horse radish peroxidase (HRP)-goat anti-mouse immunoglobulin (Invitrogen Thermo Fisher, Waltham, MA, USA, cat. # 31430) was added to the plates and incubated for 2 h at room temperature. The plates were therefore washed and incubated with 100 µL/well of the 3.3,5.5-tetramethylbenzidine substrate (SurModics BioFX, Edina, MN, USA) for 8 min at room temperature. The reaction was blocked with 50 µL/well of 1 M H_2_SO_4_. Endpoint titers were calculated as the reciprocal of the highest dilution that exceeded the cut-off, calculated as the mean absorbance of plasma from naïve mice +0.1 O.D.

### 2.5. Cell Isolation from Spleens, Blood, and the Lungs

PBMCs were recovered from the EDTA-blood samples obtained through a retro-orbital puncture under topical anesthesia. Erythrocytes were removed by treatment with ACK lysing buffer (Gibco Thermo Fisher) according to the manufacturer’s instructions.

To isolate the splenocytes, the spleens were explanted, placed into tubes containing 1 mL of RPMI 1640 and 50 µM 2-mercaptoethanol, and then transferred into 60 mm Petri dishes with 2 mL of the same medium. The splenocytes were obtained by notching the spleen sac and pushing the cells out with the plunger seal of a 1 mL syringe. After the addition of 2 mL of medium, the cells were transferred into a 15 mL conical tube, and the Petri plate was washed with 4 mL of medium to maximize cell recovery. Afterwards, cells were collected by centrifugation, resuspended in the RPMI complete medium containing 50 µM 2-mercaptoethanol and 10% FCS, and counted.

For lung cell isolation, circulating blood cells were fluorescently labeled with 2 μg (10 µL of the commercial stock) of an anti-CD45 antibody (Tonbo-Bioscience, S. Diego, CA anti-mouse CD45.2-APC, cat. 20-04540U100) diluted in 200 µL of 1× PBS inoculated in the tail vein exactly 3 min before cervical dislocation. For cell recovery, the lungs were excised, washed with 1× PBS, cut into small pieces, and then digested for 30 min under gentle agitation at 37 °C with 7 mL of 4 mg/mL of type III collagenase (Worthington Biochemical, Lakewood, NJ, USA) and 0.05 mg/mL of DNase I (Sigma) in 1× PBS. After digestion, an equal volume of medium was added, and the cells were passed through a 70 µm cell strainer, washed, and resuspended in 1× PBS/ACK 1:1 for red blood cell lysis. Finally, the isolated cells were resuspended in a complete culture medium and counted.

### 2.6. IFN-γ ELISpot Analysis

A total of either 2.5 × 10^5^ for splenocytes or 10^5^ for PBMCs live cells were seeded in triplicate in microwells of 96-multiwell plates (Millipore, Burlington, MA, USA) previously coated with the anti-mouse IFN-γ AN18 mAb (Mabtech, Nacka Strand, Sweden) in RPMI 1640, 10% heat-inactivated fetal calf serum (FCS, Gibco, Thermo Fisher), and 50 μM 2-mercaptoethanol. Cell cultures were carried out for 16 h in the presence of 5 μg/mL of the following CD8-specific, H2b-binding, SARS-CoV-2-specific peptides: Spike_539–546_: VNFNFNGL [15], M_173–180_: RTLSYYKL [16], and N_219–228_: ALALLLLDRL [16]. For the negative controls, 5 μg/mL of unrelated H2b-binding peptides were used. Peptide preparations were obtained from the BEI resources. To check for cell responsiveness, 10 ng/mL phorbol 12-myristate 13-acetate (PMA, Sigma) plus 500 ng/mL of ionomycin (Sigma) were added to the cultures. After 16 h, the cells were discarded, and the plate was incubated for 2 h at room temperature with R4-6A2 biotinylated anti-IFN-γ antibody (Mabtech) at the concentration of 100 μg/mL. The wells were then washed and treated for 1 h at room temperature with 1:1000 diluted streptavidin-ALP from Mabtech. Afterwards, 100 μL/well of SigmaFast BCIP/NBT were added to the wells to develop spots. Spot-forming units (SFUs) were finally analyzed and counted using an AELVIS ELISpot reader (Hannover, Germany).

### 2.7. Intracellular Cytokine Staining (ICS) and Flow Cytometry Analysis

The cells were cultured at 1 × 10^7^/mL in the RPMI medium, 10% FCS, 50 µM 2-mercaptoethanol (Sigma), 1 µg/mL of Brefeldin A (BD Biosciences, Franklin Lakes, NJ, USA), and in the presence of 5 μg/mL of either the spike-, N-, or unrelated H2-b CD8^+^ T-specific peptides. Positive controls were conducted by adding 10 ng/mL of PMA (Sigma) plus 1 µg/mL of ionomycin (Sigma). After 16 h, the cells were stained with 1 µL of LIVE/DEAD Fixable FVD-eFluor506 Dead Cell reagent (Invitrogen Thermo Fisher) in 1 mL of 1× PBS for 30 min at 4 °C, and the excess dye was removed by 2 washes with 500 µL of 1× PBS. Non-specific staining was minimized by pre-incubating the cells with 0.5 µg of Fc-blocking mAbs (i.e., anti-CD16/CD32 antibodies, Invitrogen/eBioscience Thermo Fisher, cat. 12-7021-82) in 100 µL of 1× PBS with 2% FCS for 15 min at 4 °C. Staining for cell surface markers was performed upon incubation for 1 h at 4 °C with 2 µL of the following anti-mouse Abs (Table 1): FITC-conjugated anti-CD3 (clone 17A2, cat. 555274, BD Biosciences), APC-Cy7-conjugated anti-CD8a (clone53-6.7, cat. 557654, BD Biosciences), PerCP-conjugated anti-CD4 (clone RM4-5, cat. 553052, BD Biosciences), and BUV395-conjugated anti-CD44 (clone IM7, cat. 740215, BD Biosciences). For ICS, the cells were fixed and permeabilized using the Cytofix/Cytoperm kit (BD Biosciences) according to the manufacturer’s recommendations. Thereafter, the cells were labeled for 1 h at 4 °C with 2 µL of the following Abs: PE-Cy7-conjugated anti-IFN-γ (eBioscience, Thermo Fisher, clone XMG1.2, cat. 25-7311-82), PE-conjugated anti-IL-2 (Invitrogen/eBioscience Thermo Fisher, clone JES6-5H4, cat. 12-7021-82), and BV421 rat anti-TNF-α (BD Biosciences, clone MP6-XT22, cat. 563387) in a total of 100 µL of 1× Perm/Wash Buffer (BD Biosciences). After two washes, the cells were fixed in 200 µL of 1× PBS/formaldehyde (2% *v*/*v*). Samples were then acquired by a CyotFLEX LX (Beckman Coulter, Brea, CA, USA) flow cytometer and analyzed using the Kaluza software 2.1 (Beckman Coulter). The gating strategy was as follows: live cells as assessed by LIVE/DEAD dye vs. FSC-A, singlet cells from FSC-A vs. FSC-H (singlet 1) and SSC-A vs. SSC-W (singlet 2), CD3^+^ cells from CD3-FITC vs. SSC-A, CD8^+^ cells from CD8-APC-Cy7 vs. CD4-PerCP. The CD3^+^/CD8^+^ cell population was gated against CD44^+^ cells, and to detect polyfunctional CD8^+^ T-lymphocytes, the population of cells positive for both CD8 and CD44 was analyzed for APC-Cy7, PE, and BV421 to detect the simultaneous changes in IFN-γ, IL-2, and TNF-α production, respectively.

### 2.8. Virus Production

Following the institutional guidelines, all virus manipulations were carried out in BSL3 conditions. VERO-E6 cells were grown in DMEM (Gibco, Thermo Fisher), supplemented with 2% FCS, 100 units/mL penicillin, 100 μg/mL streptomycin, 2 mM L-glutamine, 1 mM sodium pyruvate, and non-essential amino acids (Gibco, Thermo Fisher). The ancestral SARS-CoV-2/Italy INMI1#52284 viral isolate (GenBank sequence accession number: MT066156) was propagated by inoculation of 70% confluent VERO-E6 cells. The infected cell culture supernatant was harvested at 72 h post-infection, clarified, aliquoted, and stored at −80 °C.

### 2.9. Mouse Infection

Before experimental infection, the mice were anesthetized with a combination of ketamine (50 mg/kg of body weight) and medetomidine (1 mg/kg of body weight) administered intraperitoneally. Thirty µL of 1:6 diluted supernatant from infected Vero-E6 cells were administered intranasally (i.n.) at 15 μL per nostril, dropwise. After the virus challenge, an intraperitoneal injection of atipamezole (1 mg/kg of body weight) was used as a reversal agent. The in vitro titration of the SARS-CoV-2/Italy INMI1#52284 isolate we used was already described [17]. A virus challenge was performed using a virus dose equivalent to 4.4 lethal doses 50% of the population (LD_50_), resulting in a 99.99%-predicted probability of mortality [17].

### 2.10. Extraction and Purification of Lung RNA

The lungs were stored frozen after excision. After thawing, equal amounts of tissues were minced and incubated for 10 min in 1 mL of TRIzol™ Reagent (Thermo Fisher Scientific). The minced tissue was then passed through a QIAshredder homogenizer (Qiagen, Germantown, MD, USA), and the flow-through was used for chloroform extraction, according to the TRIzol™ protocol, using 0.2 mL of chloroform. The recovered total RNA was stored in water at −80 °C.

### 2.11. RT-qPCR

RT-qPCR for the SARS-CoV-2 Envelope (E) and Nucleocapsid (N) genes was performed using a one-step TaqMan-based strategy, as previously described [13]. Mouse β-actin amplification was included as the loading control. All probes and primers were purchased from Integrated DNA Technologies (IDT, Leuven, Belgium). Each 20 µL reaction mixture contained 12 μL of the qPCRBIO Probe 1-Step Virus Detect Lo ROX master mix (PCR Biosystems Ltd., London, UK), 3 µL of the primers/probes mix, and 1 µg of ezDNAse (Thermo Fisher)-treated RNA in a total of 5 µL. All samples were tested in duplicate, and the samples with nuclease-free water alone were included as negative controls. Serial 10-fold dilutions of E gene plasmid (10006896, 2019-nCoV_E Positive Control from Charité/Berlin, IDT) and N1/N2 plasmid (10006625, 2019-nCoV_N_Positive Control from CDC, IDT) were used to generate standard curves ranging from 1 to 10^5^ copies. The median standard curve slope ranged from 3.25 to 3.43, with R^2^ > 0.998.

The samples were run on an Applied Biosystems 7500 Fast PCR system (Thermo Fisher). The following cycling conditions were applied: reverse transcription for 10 min at 55 °C, followed by denaturation at 95 °C for 3 min. Then, 50 cycles of denaturation at 95 °C for 15 s and annealing/extension at 58 °C for 30 s. Amplification data were analyzed using the Applied Biosystems 7500 software, v2.3 (Thermo Fisher). The results are reported as numbers of RNA copies for the μg of total RNA. The experimental limits of detection, determined by Probit analysis, were 6.76 copies/reaction and 5.85 copies/reaction for the E and N transcripts, respectively.

### 2.12. Statistical Analysis

Whenever possible, data are presented as the mean values ± standard error (SE). When appropriate, the one- or two-tailed Mann–Whitney U tests, the two-tailed Student’s t-test with the Welch correction, or the Kruskal–Wallis test followed by Dunn’s multiple comparisons test were conducted. Statistical analyses and graphs were generated with GraphPad Prism 9.5.1 software (GraphPad 9.5.1). A *p* < 0.05 was considered significant.

## 3. Results

### 3.1. Induction of SARS-CoV-2-Specific CD8^+^ T Cells in Mice Infected Shortly after Vaccine Boosting

**Figure 1:**
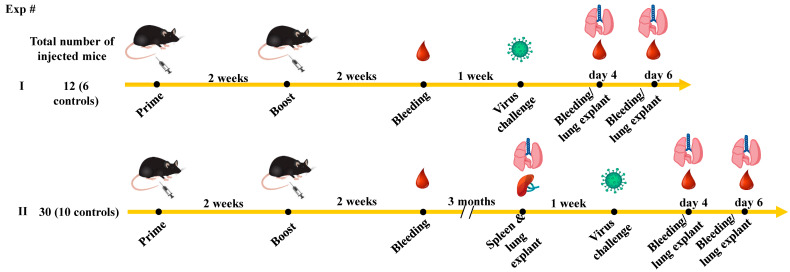
Scheme depicting both flow and time points of the immunization/infection experiments carried out in K18-hACE2 mice. Also shown is the number of animals used in both experiments.

Mice (6 per group) were injected with either the spike-expressing or unrelated DNA vectors twice, 2 weeks apart (Figure 1).

**Figure 2:**
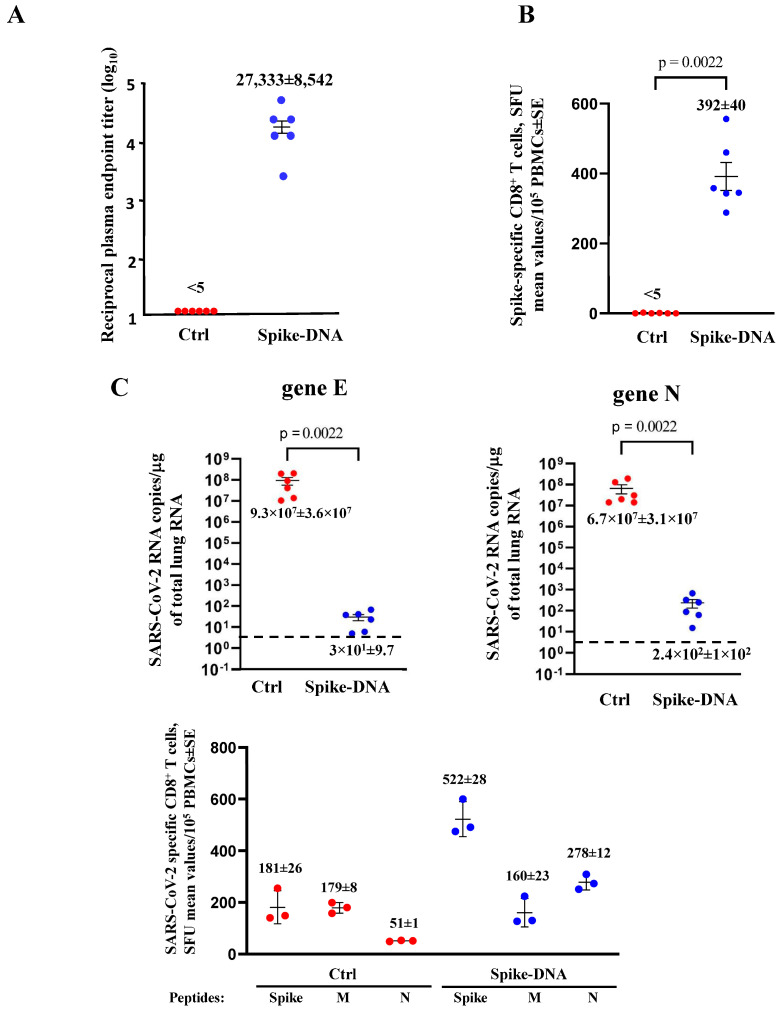
Spike-specific immune responses and viral replication in spike-DNA injected mice. (**A**) Detection of anti-S1 antibodies in plasma of K18-hACE2 mice i.m. injected with either void (Ctrl, 6 mice) or spike-expressing (6 mice) DNA vectors. The log_10_ of reciprocal endpoint titers is shown, together with intragroup mean ± SE. (**B**) Detection of SARS-CoV-2-spike-specific CD8^+^ T-cells in PBMCs. A total of 10^5^ PBMCs were incubated overnight with 5 μg/mL of either unrelated or spike-specific peptide in IFN-γ ELISpot microwells. The numbers are shown of spot-forming units (SFUs)/well calculated as mean values of triplicates after subtraction of the mean spot numbers detected in wells of PBMCs treated with an unspecific peptide (<5 spots for each cell culture tested). The intragroup mean values ±SE are reported. (**C**) Viral loads in the lungs of immunized/infected mice. Four and six days after the challenge, injected mice (6 per group) were sacrificed, and the lungs were processed for the extraction of total RNA. Then, 1 μg of total RNA from each infected mouse was analyzed by RT-qPCR for the presence of both SARS-CoV-2 E- and N-specific RNAs. The numbers are shown of both E and N viral RNA copies, amplified from total RNA isolated from the lungs of each animal, together with intragroup mean values ± SE. Dotted lines signal the detection limits of the assay. (**D**) Detection of spike-, M-, and N-specific CD8^+^ T-cells in PBMCs isolated 6 days after infection. A total of 10^5^ PBMCs were incubated overnight with or without 5 μg/mL of either unrelated spike-, M-, or N-specific peptides in IFN-γ ELISpot microwells. The numbers of SFUs/well calculated are shown as mean values of triplicates after subtraction of the mean SFUs detected in wells of PBMCs treated with unspecific peptides (<4 spots for each cell culture tested). The intragroup mean values ±SE are reported.

After an additional two weeks, the anti-spike antibodies (Abs) in plasma were measured by ELISA, and the peripheral blood mononuclear cells (PBMCs) were tested for the presence of spike-specific CD8^+^ T-cells by ELISpot assay. The immunized mice showed a strong response in terms of both induction of anti-spike Abs (Figure 2A) and spike-specific CD8^+^ T-cells (Figure 2B). One week later, the mice were intranasally infected with 4.4 LD_50_ of SARS-CoV-2, and the viral replication levels in the lungs were assessed 4–6 days after the challenge. Viral spread in the lungs appeared dramatically hampered in vaccinated mice, with a mean reduction of more than 5 logs (Figure 2C) in the absence of weight loss (Supplementary Figure S1).

The PBMCs from infected mice were isolated at 4 and 6 days after infection, and the presence of the SARS-CoV-2 spike-, M-, and N-specific CD8^+^ T-cells was evaluated by ELISpot analysis. At day 4 after infection, spike-specific CD8^+^ T-cells were found in vaccinated mice only, while both the M- and N-specific CD8^+^ T-cells were undetectable (Supplementary Figure S2). At day 6 post-infection, there was a sustained spike-specific CD8^+^ T-cell immune response in vaccinated mice coupled with the appearance of both the M- and N-CD8^+^ T-cells, whose levels did not decline compared to the control mice (Figure 2D).

**Figure 3:**
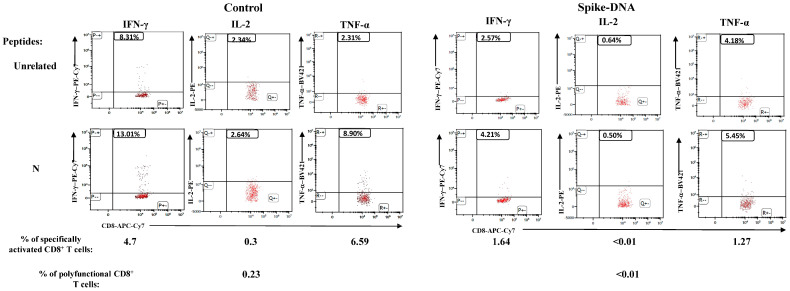
SARS-CoV-2 N-specific CD8^+^ T-lymphocytes in cells isolated from the lungs of both unvaccinated and vaccinated K18-hACE2 mice after infection. Representative dot plots are shown from ICS/flow cytometry analysis for the detection of IFN-γ, IL-2, and TNF-α within the CD45^-^ fraction of cells isolated from the lungs and cultivated in the presence of either unrelated or N-specific peptides. Percentages of specifically activated as well as polyfunctional CD8^+^ T-cells are indicated.

When the investigation was extended to the immune cells isolated from the lungs, it appeared that six days after infection, the N-specific CD8^+^ T-cell immune response in vaccinated/infected mice was lower compared to that detected in the control’s infected mice, as measured in terms of the intracellular accumulation of IFN-γ, IL-2, and TNF-α (Figure 3). Polyfunctional CD8^+^ T-cells (i.e., cells co-expressing the three cytokines) were barely detectable in the unvaccinated mice only. Of note, due to the limited number of cells recovered from the lungs, the results were obtained from duplicate cultures where cells from the three available infected mice were pooled.

We concluded that, shortly after boosting, the levels of virus-specific CD8^+^ T-cell immunity, as detected in circulatory cells, were not influenced by the vaccination; however, this led to a scarce diffusion of antiviral CD8^+^ T-cells in the lungs.

### 3.2. SARS-CoV-2 Infection Induces Circulatory Virus-Specific CD8^+^ T-Cells at Similar Levels in Unvaccinated Mice and in Mice Three Months after Vaccine Boost

Electroporation procedures have the potential to induce both localized and transient immune activation [18]. To extend and validate our observations as well as to exclude possible biases in the interpretation of data, the vaccination/infection experiments were reproduced by also immunizing the mice with the mRNA-based, commercial Spikevax vaccine and challenging the mice three months after the vaccine boosts (Figure 1).

**Figure 4:**
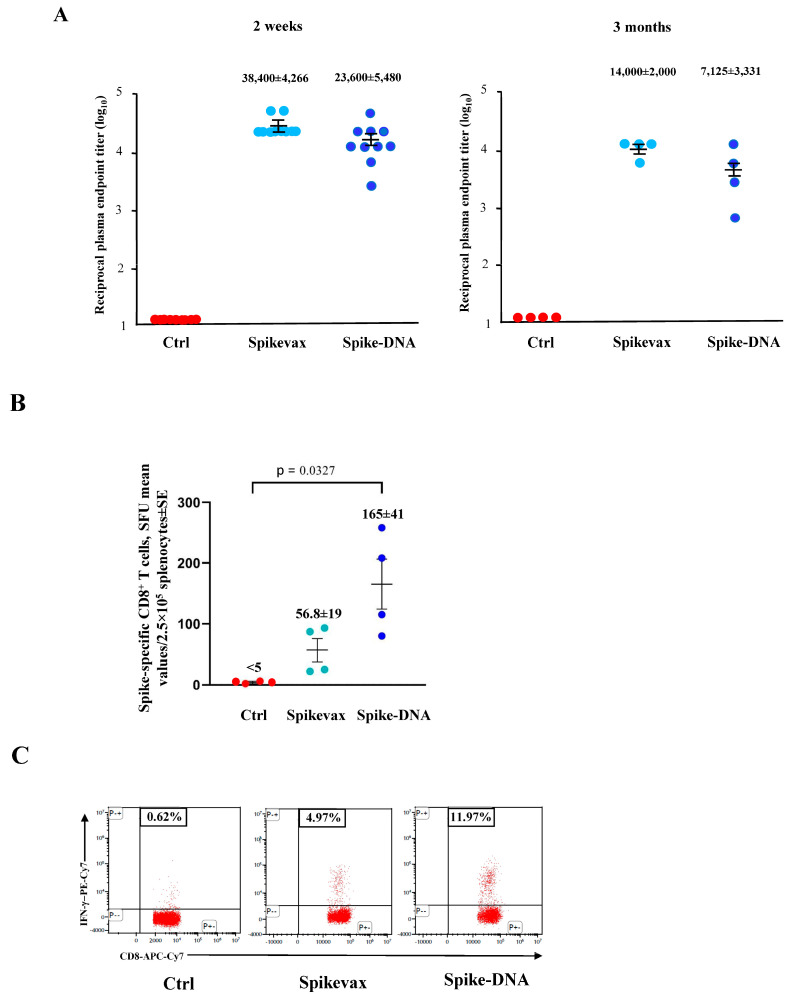
Spike-specific immune responses in both Spikevax and DNA-spike vaccinated mice. (**A**) Detection of anti-S1 antibodies in plasma of K18-hACE2 mice two weeks (on the left) and three months (on the right) after injection with either Spikevax or Spike-DNA vaccines. As a control, mice were injected with isotonic buffer (Ctrl). Measures were conducted in plasma from 10 (two weeks after boosting) and 4 (three months after boosting) mice per group. The log_10_ of reciprocal endpoint titers is shown, together with intragroup means ± SE. (**B**) Detection of SARS-CoV-2-spike-specific CD8^+^ T-cells in splenocytes isolated from K18-hACE2 mice i.m. injected with either Spikevax vaccine, spike-expressing DNA vector, or buffer (Ctrl, 4 mice per group) three months after vaccination. A total of 2.5 × 10^5^ splenocytes were incubated overnight with 5 μg/mL of either unrelated or spike-specific peptide in IFN-γ ELISpot microwells. The numbers of SFUs/well calculated are shown as mean values of triplicates after subtraction of the mean SFUs counted in wells with splenocytes treated with the unspecific peptide (<10 spots for each cell culture tested). The intragroup mean values ±SE are reported. (**C**) Representative dot plots from ICS/flow cytometry analysis for the expression of IFN-γ in PBMCs from mice injected, as indicated. Cells were cultivated overnight with either a spike-specific or an unrelated peptide. Data shown were obtained with PBMCs pooled from two mice per condition, representative of two experiments. Quadrants were set on the basis of cell fluorescence of samples treated with the unrelated peptide.

High levels of anti-spike Abs were found in immunized mice (10 per group) both two weeks and three months after boosting (Figure 4A), with a partial waning over time. On the other hand, circulatory spike-specific CD8^+^ T-cells were still detectable by both ELISpot and ICS/flow cytometry assays (Figure 4B,C). Mice were then infected as described above, and the SARS-CoV-2 replication levels were evaluated by reverse-transcriptase (RT)-qPCR, carried out on the total RNA extracted from the lungs 4–6 days after infection.

**Figure 5:**
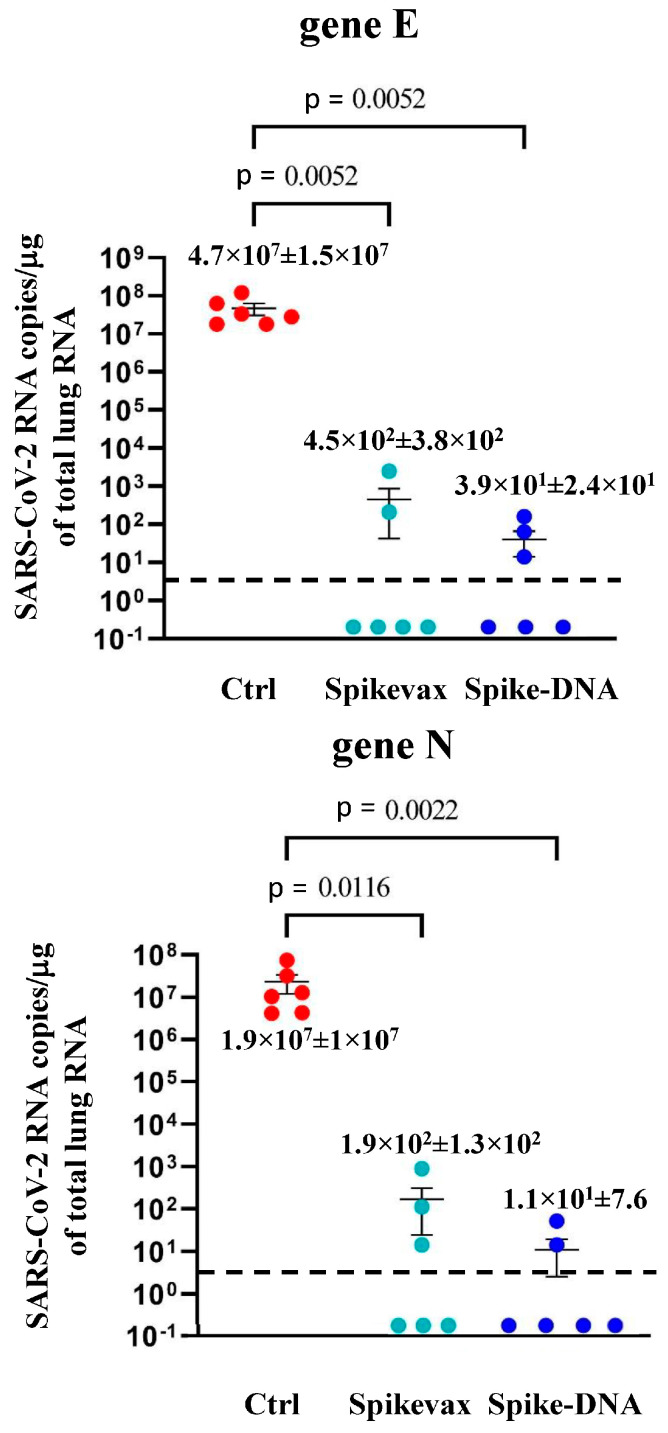
Viral loads in the lungs of mice infected three months after boosting. Four to six days after the challenge, mice (6 per group) were sacrificed, and the lungs were processed for the extraction of total RNA. Then, 1 μg of total RNA from each infected mouse was analyzed by RT-qPCR for the presence of both SARS-CoV-2 E- and N-specific RNAs. Shown are the numbers of E- and N-specific viral RNA copies amplified from total RNA isolated from the lungs of each animal, together with intragroup mean values ±SE. Dotted lines signal the detection limits of the assay.

We noticed more than a 5-log reduction of the virus’s replication in both the Spikevax- and DNA-immunized mice (Figure 5). No weight loss was observed in the vaccinated/infected mice (Supplementary Figure S3).

**Figure 6:**
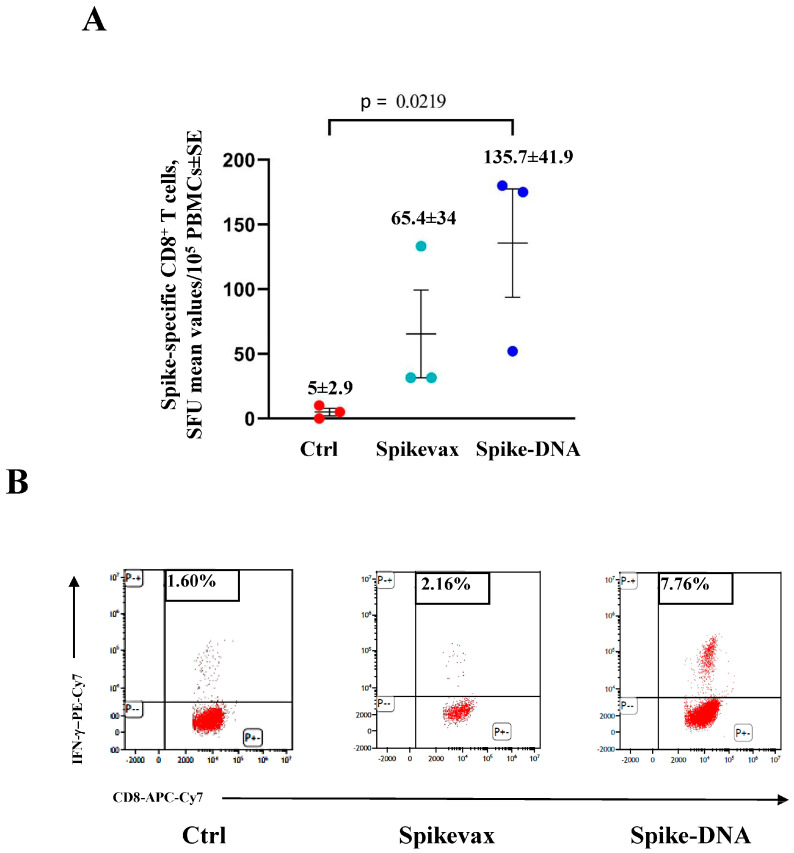
Induction of spike-specific CD8^+^ T-cells in K18-hACE2 mice three months after vaccine boosting and 4 days after infection. (**A**) Data from ELISpot analysis. A total of 10^5^ PBMCs isolated from infected and either vaccinated or unvaccinated mice (3 per group) were incubated overnight with 5 μg/mL of either unrelated or spike-specific peptide in IFN-γ ELISpot microwells. The numbers of SFUs/well calculated are shown as mean values of triplicates after subtraction of the mean SFUs detected in wells where PBMCs were treated with an unspecific peptide (<3 spots for each cell culture tested). The intragroup mean values ±SE are reported. (**B**) Representative dot plots from ICS/flow cytometry analysis for the expression of IFN-γ in PBMCs. Cells were cultivated overnight with either the spike or an unrelated peptide. The results obtained with PBMCs pooled from three mice per condition are reported. Quadrants were set on the basis of cell fluorescence of samples treated with an unrelated peptide.

Next, the induction of both the SARS-CoV-2 M- and N-specific CD8^+^ T-cells was monitored by ELISpot and ICS/flow cytometry analyses of the PBMCs isolated 4 and 6 days after infection. At day 4 post-infection, both the M- and N-specific CD8^+^ T-cell lymphocytes remained undetectable in all infected mice (Supplementary Figure S4) in the presence, as expected, of spike-specific CD8^+^ T-cells in vaccinated mice (Figure 6).

**Figure 7:**
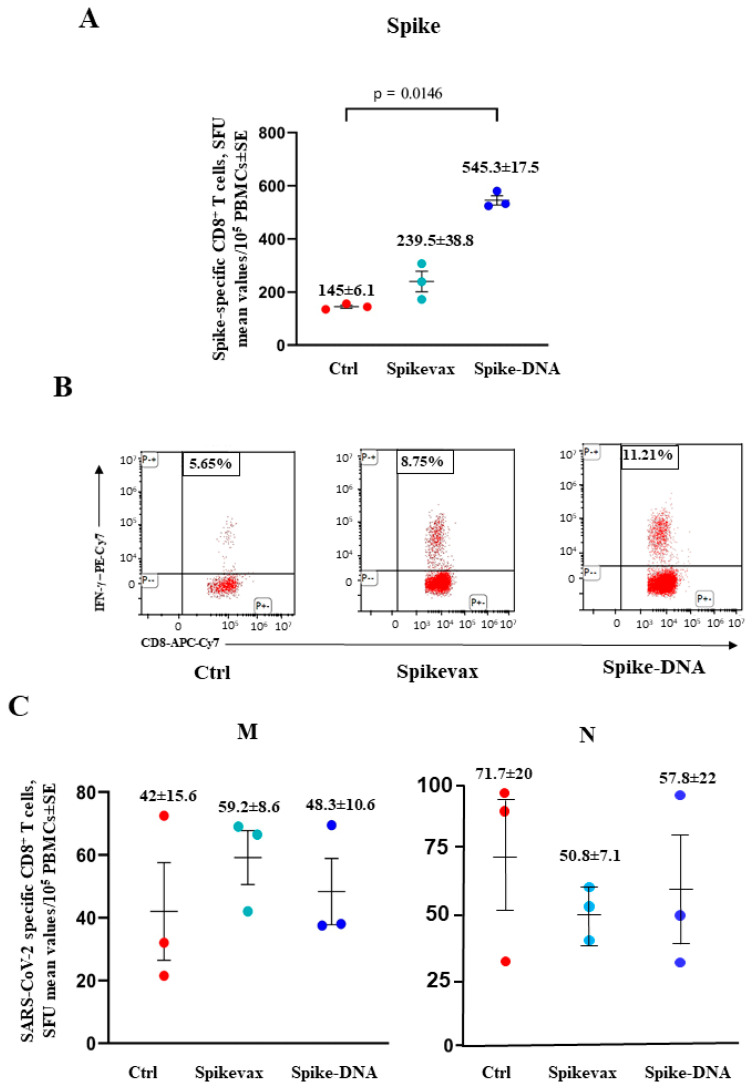
Induction of SARS-CoV-2-specific CD8^+^ T-cells in K18-hACE2 mice three months after boosting and 6 days after infection. (**A**) Detection of spike-specific CD8^+^ T-cells in both vaccinated and unvaccinated mice (3 per group). A total of 10^5^ PBMCs were incubated overnight with 5 μg/mL of either unrelated or spike-specific peptide in IFN-γ ELISpot microwells. The numbers of SFUs/well calculated are shown as mean values of triplicates after subtraction of the mean SFUs detected in wells of PBMCs treated with an unspecific peptide (<7 spots for each cell culture tested). The intragroup mean values ± SE are reported. (**B**) Representative dot plots from ICS/flow cytometry analysis for the expression of IFN-γ in PBMCs are reported. Cells were cultivated overnight with either a spike-specific or an unrelated peptide. Data shown were obtained with PBMCs pooled from three mice per condition. Quadrants were set on the basis of cell fluorescence of samples treated with the unrelated peptide. (**C**) Detection of both M- and N-specific CD8^+^ T-cells in PBMCs isolated 6 days after infection. A total of 10^5^ PBMCs were incubated overnight with or without 5 μg/mL of either unrelated, M- or N-specific peptides in IFN-γ ELISpot microwells. The numbers of SFUs/well calculated are shown as mean values of triplicates after subtraction of mean SFUs calculated in wells where PBMCs were treated with unspecific peptides (<3 spots for each cell culture tested). Intragroup mean values ± SE are reported.

Six days after infection, the percentages of spike-specific CD8^+^ T-cells increased significantly in both groups (Figure 7A,B). Most notably, at this time, both the M- and N-specific CD8^+^ T-cells became readily detectable at similar levels in the vaccinated and unvaccinated mice (Figure 7C).

These results further supported the idea that the virus-specific CD8^+^ T-cell immune response after infection can be induced in blood, irrespective of the vaccination state.

### 3.3. Lack of De Novo Induction of SARS-CoV-2-Specific CD8^+^ T-Cells in the Lungs of Mice Infected Three Months after Vaccine Boost

An ICS/flow cytometry-based assay was performed on the cells isolated from the lungs of mice before and after the virus challenge, which was carried out three months after the vaccine boost. In detail, lung cells were analyzed seven days before infection, as well as 4 and 6 days after the challenge by pooling the cells isolated from two/three mice per group. The most evident outcome was the presence of both the spike- and N-specific CD8^+^ T-cells in control infected mice, while the infection was not associated with the de novo induction of SARS-CoV-2-specific CD8^+^ T-cells in the vaccinated ones, as indicated by the lack of N-specific cell immunity (Table 2). Notably, polyfunctional CD8^+^ T-cells have been found in unvaccinated mice only.

These data were consistent with what was observed in mice infected soon after vaccination and strongly suggested that in the vaccinated mice, CD8^+^ T-cell-specific antiviral immunity in the lungs benefits poorly from the breakthrough infection.

## 4. Discussion

BTI potently strengthens the systemic anti-SARS-CoV-2 immunity, including the induction of virus-specific CD4^+^ and CD8^+^ T-cells in both blood and the nasal cavity [19,20]. However, less is known concerning the diffusion of virus-specific immune cells in the lungs. Shedding light on such a still unexplored issue would be of utility for the interpretation of the dynamics of the induction of CD8^+^ T-cell immunity in vaccinated/infected people, as well as for the development of new mucosal T-cell-based anti-SARS-CoV-2 vaccines.

After the intranasal infection of K18-hACE2 mice, the virus diffuses quite rapidly; thus, similar amounts of infectious viral particles can be found in both nasal turbinate and the lungs as early as 24 h post-challenge [12,21]. The viral load gradually decreases in nasal turbinate, while it peaks at day 2 post-infection in the lungs, where it persists at high levels until days 6–7 post-infection [22]. The vaccination protocols we applied appeared quite effective in reducing the viral load in the lungs of infected mice, and the overlapping data we obtained three weeks and three months after vaccine boosting excluded possible biases due to a general transient immune hyperactivation consequence of electroporation procedures [18] and/or DNA loading. Additional confounding effects not related to the spike-specific DNA vaccination can also be excluded, considering the similar results we obtained with the mRNA-based vaccine.

When we investigated whether the vaccination influenced the induction of circulatory SARS-CoV-2-specific CD8^+^ T-cells, we did not find major differences between the control and vaccinated mice apart from, as expected, the high levels of spike-specific CD8^+^ T-lymphocytes in vaccinated animals, consistent with what was observed in humans after a BTI [19]. De novo SARS-CoV-2-specific circulatory CD8^+^ T-cells appeared not before six days after the challenge, irrespective of the vaccination status.

Differently, when observations were conducted on the immune cells isolated from the lungs of challenged mice, we reproducibly found that the infection in vaccinated mice was not associated with the presence of the high levels of virus-specific CD8^+^ T-cells observed in unvaccinated infected mice. Of note, and different from what was observed in humans [10], spike-specific CD8^+^ T-cells populated the lungs of vaccinated mice before infection. Most likely, this difference was due to the excess dose/kg of vaccine used compared to the standard dose injected into humans, e.g., from 10- to 40-fold higher in the case of the mRNA-based vaccine.

The finding that the virus-specific CD8^+^ T-cells appearing in the blood of vaccinated mice after infection diffused poorly to the lungs was consistent with the already demonstrated evidence that circulatory immune cells permeate the lung tissues inefficiently, in line with the concept that the lung immune system is compartmentalized [23]. One can hypothesize that in the presence of the block of the virus spread to the lungs dictated by vaccination, the immunogenic stimulus generated by the infection was not strong enough to induce the diffusion of circulatory SARS-CoV-2-specific CD8^+^ T-cells towards the lung tissues.

Our study has a number of limitations: the experimental groups included a limited number of mice, and the viral replication was evaluated in the lungs only. However, there is evidence that the viral replication in vaccinated mice was inhibited at similar extents in the lungs, turbinate, and oral cavity [24,25]. Furthermore, due to the limited number of recovered cells, statistics cannot be applied to the results obtained by the ICS/flow cytometry analysis of the immune cells isolated from the lungs. The reliability of these data, however, is supported by the consistent outcomes we obtained with the lung immune cells isolated three weeks and three months after vaccine boosting.

Our findings would help in deciphering the actual effects of a BTI in terms of induction of antiviral cell immunity. In addition, these results indicate that, for complete protection, innovative anti-SARS-CoV-2 mucosal vaccines need to target the lung tissues. Consistent with this idea, the superiority of the aerosol versus intranasal administration of the same vaccine formulation has been recently demonstrated in a model of anti-flu vaccines in pigs [26]. Taking advantage of the presented data, we are exploring an innovative mucosal vaccine based on the antiviral CD8^+^ T-cell immunity induced by extracellular vesicles engineered for the incorporation of SARS-CoV-2 N protein [13].

## 5. Conclusions

In sum, here, we presented the results that support the idea that a BTI does not efficiently induce antiviral cell immunity in the lungs. Despite some limitations, the K18-hACE2 mouse can be considered a reliable model for the study of SARS-CoV-2-related pathogenesis and vaccines. However, our findings need to be confirmed in alternative animal models and, most importantly, in humans using, for instance, bronchoalveolar lavage fluids from vaccinated/infected subjects.

## Figures and Tables

**Table 1:** Antibodies used for flow cytometry analyses.

Mouse Target	Clone	Conjugation	Company	Working Dilution
CD3	17A2	FITC	BD	1:50
CD8a	53-6.7	APC-Cy7	BD	1:100
CD4	RM4-5	PerCP	BD	1:100
CD44	IM7	BUV395	BD	1:50
IFN-γ	XMG1.2	PE-Cy7	eBioscence	1:50
IL-2	JES6-5H4	PE	eBioscence	1:100
TNF-α	MP6-XT22	BV421	BD	1:100

**Table 2:** Detection of SARS-CoV-2 spike- and N-specific CD8^+^ T-lymphocytes from the lungs of both unvaccinated and vaccinated K18-hACE2 mice before and after infection ^a^.

	Control				Spike-DNA			
Peptide	Spike		N		Spike		N	
Days	IFN-γ^+^	Triple	IFN-γ^+^	Triple	IFN-γ^+^	Triple	IFN-γ^+^	Triple
−7	<0.01	<0.01	<0.01	<0.01	4.30	1.49	<0.01	<0.01
+4	6.36	4.36	2.35	1.26	5.21	1.01	<0.01	<0.01
+6	7.11	1.86	0.62	0.12	1.71	<0.01	<0.01	<0.01

The percentages of cells expressing IFN-γ and co-expressing IFN-γ, IL-2, and TNF-α over the total of CD8^+^/CD44^+^ T-cells within lung immune cells pooled from three/four mice, either unvaccinated or vaccinated, with spike-expressing DNA. The mean values of the percentages of cytokine-expressing cells from duplicated pooled cell cultures treated with either spike or N peptides after subtraction of values detected in cultures treated with an unrelated peptide. Detections were made 7 days before infection and both 4 and 6 days after challenge.

## Data Availability

The data presented in this study are available on request from the corresponding author.

## Supplementary Materials

The following supporting information can be downloaded at https://www.mdpi.com/article/10.3390/vaccines11091433/s1, Figure S1: Relative weight loss in mice infected by SARS-CoV-2 three weeks after vaccine booster. Figure S2: Detection of spike-, M, and N-specific CD8^+^ T-cells in PBMCs isolated 4 days after infection. Figure S3: Relative weight loss in mice infected by SARS-CoV-2 three months after vaccine booster. Figure S4: Detection of both M-and N-specific circulatory CD8^+^ T-cells in K18-huACE2 mice three months after vaccine boosting and 4 days after infection.

## References

[1] Federico M. (2021). Virus-Induced CD8+ T-Cell Immunity and Its Exploitation to Contain the SARS-CoV-2 Pandemic. Vaccines.

[2] Guo L., Wang G., Wang Y., Zhang Q., Ren L., Gu X., Huang T., Zhong J., Wang Y., Wang X. (2022). SARS-CoV-2-Specific Antibody and T-Cell Responses 1 Year after Infection in People Recovered from COVID-19: A Longitudinal Cohort Study. Lancet Microbe.

[3] Kundu R., Narean J.S., Wang L., Fenn J., Pillay T., Fernandez N.D., Conibear E., Koycheva A., Davies M., Tolosa-Wright M. (2022). Cross-Reactive Memory T Cells Associate with Protection against SARS-CoV-2 Infection in COVID-19 Contacts. Nat. Commun..

[4] McMahan K., Yu J., Mercado N.B., Loos C., Tostanoski L.H., Chandrashekar A., Liu J., Peter L., Atyeo C., Zhu A. (2021). Correlates of Protection against SARS-CoV-2 in Rhesus Macaques. Nature.

[5] Peng Y., Felce S.L., Dong D., Penkava F., Mentzer A.J., Yao X., Liu G., Yin Z., Chen J.-L., Lu Y. (2022). An Immunodominant NP105-113-B*07:02 Cytotoxic T Cell Response Controls Viral Replication and Is Associated with Less Severe COVID-19 Disease. Nat. Immunol..

[6] Bange E.M., Han N.A., Wileyto P., Kim J.Y., Gouma S., Robinson J., Greenplate A.R., Hwee M.A., Porterfield F., Owoyemi O. (2021). CD8+ T Cells Contribute to Survival in Patients with COVID-19 and Hematologic Cancer. Nat. Med..

[7] Puhach O., Adea K., Hulo N., Sattonnet P., Genecand C., Iten A., Jacquérioz F., Kaiser L., Vetter P., Eckerle I. (2022). Infectious Viral Load in Unvaccinated and Vaccinated Individuals Infected with Ancestral, Delta or Omicron SARS-CoV-2. Nat. Med..

[8] Garcia-Knight M., Anglin K., Tassetto M., Lu S., Zhang A., Goldberg S.A., Catching A., Davidson M.C., Shak J.R., Romero M. (2022). Infectious Viral Shedding of SARS-CoV-2 Delta Following Vaccination: A Longitudinal Cohort Study. PLoS Pathog..

[9] Kared H., Wolf A.-S., Alirezaylavasani A., Ravussin A., Solum G., Tran T.T., Lund-Johansen F., Vaage J.T., Nissen-Meyer L.S., Nygaard U.C. (2022). Immune Responses in Omicron SARS-CoV-2 Breakthrough Infection in Vaccinated Adults. Nat. Commun..

[10] Tang J., Zeng C., Cox T.M., Li C., Son Y.M., Cheon I.S., Wu Y., Behl S., Taylor J.J., Chakaraborty R. (2022). Respiratory Mucosal Immunity against SARS-CoV-2 after MRNA Vaccination. Sci. Immunol..

[11] McCray P.B., Pewe L., Wohlford-Lenane C., Hickey M., Manzel L., Shi L., Netland J., Jia H.P., Halabi C., Sigmund C.D. (2007). Lethal Infection of K18-HACE2 Mice Infected with Severe Acute Respiratory Syndrome Coronavirus. J. Virol..

[12] Morales Vasquez D., Chiem K., Silvas J., Park J.-G., Ye C., Martínez-Sobrido L. (2021). Live Imaging and Quantification of Viral Infection in K18 HACE2 Transgenic Mice Using Reporter-Expressing Recombinant SARS-CoV-2. J. Vis. Exp..

[13] Manfredi F., Chiozzini C., Ferrantelli F., Leone P., Pugliese K., Spada M., Di Virgilio A., Giovannelli A., Valeri M., Cara A. (2023). Antiviral Effect of SARS-CoV-2 N-Specific CD8+ T Cells Induced in Lungs by Engineered Extracellular Vesicles. NPJ Vaccines.

[14] Dispinseri S., Secchi M., Pirillo M.F., Tolazzi M., Borghi M., Brigatti C., De Angelis M.L., Baratella M., Bazzigaluppi E., Venturi G. (2021). Neutralizing Antibody Responses to SARS-CoV-2 in Symptomatic COVID-19 Is Persistent and Critical for Survival. Nat. Commun..

[15] Zhi Y., Kobinger G.P., Jordan H., Suchma K., Weiss S.R., Shen H., Schumer G., Gao G., Boyer J.L., Crystal R.G. (2005). Identification of Murine CD8 T Cell Epitopes in Codon-Optimized SARS-Associated Coronavirus Spike Protein. Virology.

[16] Zhao J., Zhao J., Perlman S. (2010). T Cell Responses Are Required for Protection from Clinical Disease and for Virus Clearance in Severe Acute Respiratory Syndrome Coronavirus-Infected Mice. J. Virol..

[17] Ferrantelli F., Chiozzini C., Manfredi F., Leone P., Spada M., Di Virgilio A., Giovannelli A., Sanchez M., Cara A., Michelini Z. (2022). Strong SARS-CoV-2 N-Specific CD8+ T Immunity Induced by Engineered Extracellular Vesicles Associates with Protection from Lethal Infection in Mice. Viruses.

[18] Justesen T.F., Orhan A., Raskov H., Nolsoe C., Gögenur I. (2022). Electroporation and Immunotherapy—Unleashing the Abscopal Effect. Cancers.

[19] Lim J.M.E., Tan A.T., Le Bert N., Hang S.K., Low J.G.H., Bertoletti A. (2022). SARS-CoV-2 Breakthrough Infection in Vaccinees Induces Virus-Specific Nasal-Resident CD8+ and CD4+ T Cells of Broad Specificity. J. Exp. Med..

[20] Koutsakos M., Reynaldi A., Lee W.S., Nguyen J., Amarasena T., Taiaroa G., Kinsella P., Liew K.C., Tran T., Kent H.E. (2023). SARS-CoV-2 Breakthrough Infection Induces Rapid Memory and de Novo T Cell Responses. Immunity.

[21] Kumari P., Rothan H.A., Natekar J.P., Stone S., Pathak H., Strate P.G., Arora K., Brinton M.A., Kumar M. (2021). Neuroinvasion and Encephalitis Following Intranasal Inoculation of SARS-CoV-2 in K18-HACE2 Mice. Viruses.

[22] Carossino M., Kenney D., O’Connell A.K., Montanaro P., Tseng A.E., Gertje H.P., Grosz K.A., Ericsson M., Huber B.R., Kurnick S.A. (2022). Fatal Neurodissemination and SARS-CoV-2 Tropism in K18-HACE2 Mice Is Only Partially Dependent on HACE2 Expression. Viruses.

[23] Allie S.R., Bradley J.E., Mudunuru U., Schultz M.D., Graf B.A., Lund F.E., Randall T.D. (2019). The Establishment of Resident Memory B Cells in the Lung Requires Local Antigen Encounter. Nat. Immunol..

[24] DiPiazza A.T., Leist S.R., Abiona O.M., Moliva J.I., Werner A., Minai M., Nagata B.M., Bock K.W., Phung E., Schäfer A. (2021). COVID-19 Vaccine MRNA-1273 Elicits a Protective Immune Profile in Mice That Is Not Associated with Vaccine-Enhanced Disease upon SARS-CoV-2 Challenge. Immunity.

[25] EMA/896245/2022 Committee for Medicinal Products for Human Use (CHMP) Assessment Report Spikevax Procedure No. EMEA/H/C/005791/II/0075/G. https://www.ema.europa.eu/en/documents/variation-report/spikevax-previously-covid-19-vaccine-moderna-h-c-005791-ii-0075-g-epar-assessment-report-variation_en.pdf.

[26] Vatzia E., Allen E.R., Manjegowda T., Morris S., McNee A., Martini V., Kaliath R., Ulaszewska M., Boyd A., Paudyal B. (2021). Respiratory and Intramuscular Immunization with ChAdOx2-NPM1-NA Induces Distinct Immune Responses in H1N1pdm09 Pre-Exposed Pigs. Front. Immunol..

