# Supplementary material for: SARS-CoV-2-Specific CD8+ T-Cells in Blood but Not in the Lungs of Vaccinated K18-hACE2 Mice after Infection
Source: Vaccines (Basel). 2023 Aug 30;11(9):1433. doi: 10.3390/vaccines11091433 (PMC10535545; doi:10.3390/vaccines11091433)
Supplement: Supplementary file 1 [file vaccines-11-01433-s001.zip › vaccines-2557653-supplementary.pdf]

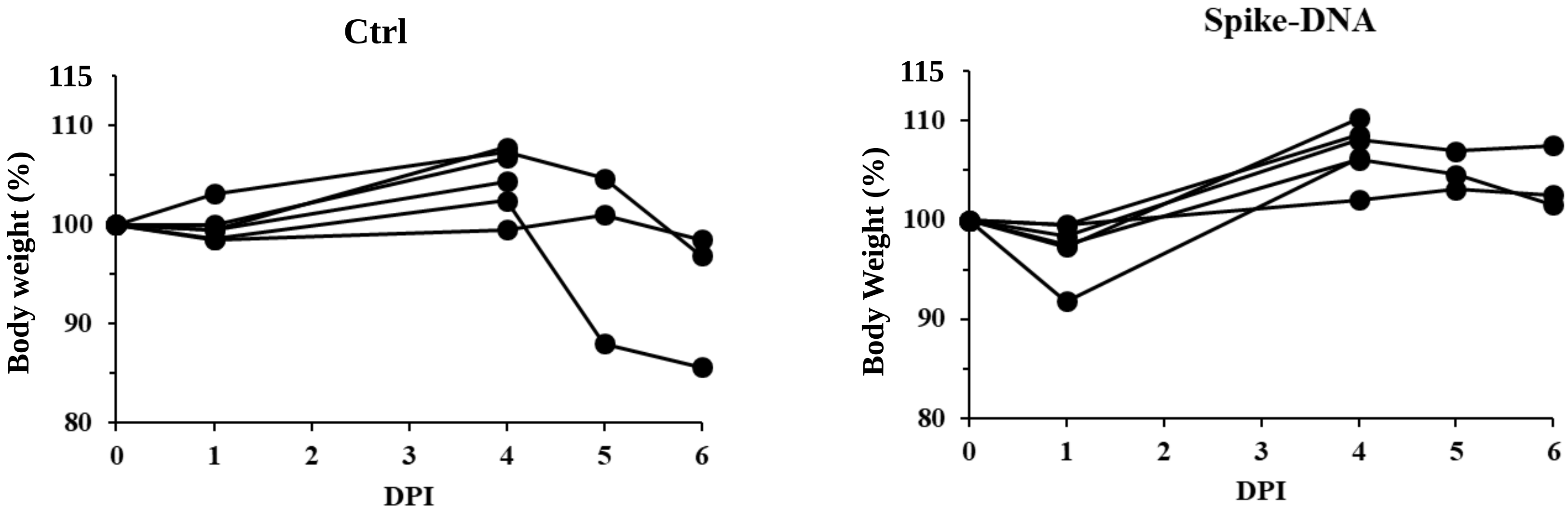

Supplementary Figure S1. Relative weight loss in mice infected by SARS-CoV-2 three weeks after vaccine booster. DPI: days post-infection.

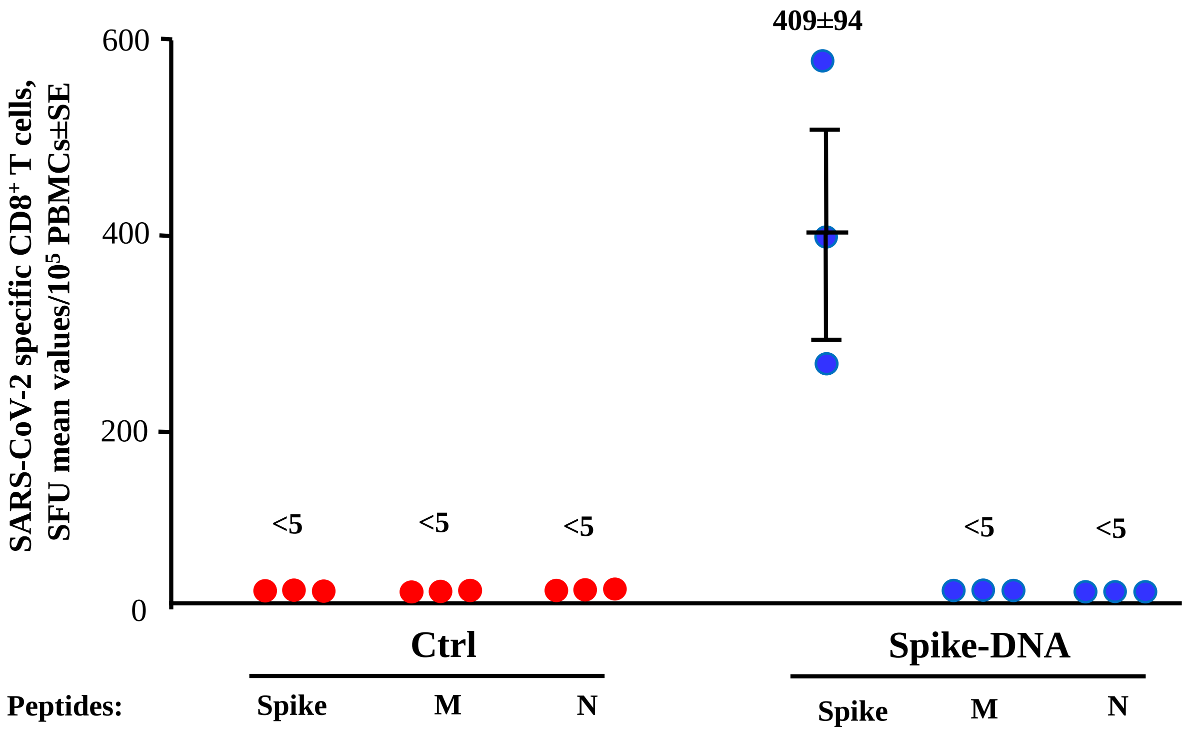

Supplementary Figure S2. Detection of Spike-, M-, and N-specific $CD8^+$ T cells in PBMCs isolated 4 days after infection. A total of 10^5 PBMCs were incubated overnight with or without 5 μg/ml of either unrelated, Spike-, M-, or N-specific peptides in IFN-γ EliSpot microwells. Shown are the numbers of SFUs/well calculated as mean values of triplicates after subtraction of the mean SFUs calculated in wells of PBMCs treated with unspecific peptides (<5 for each cell culture tested). Reported is intragroup mean value ±SE.

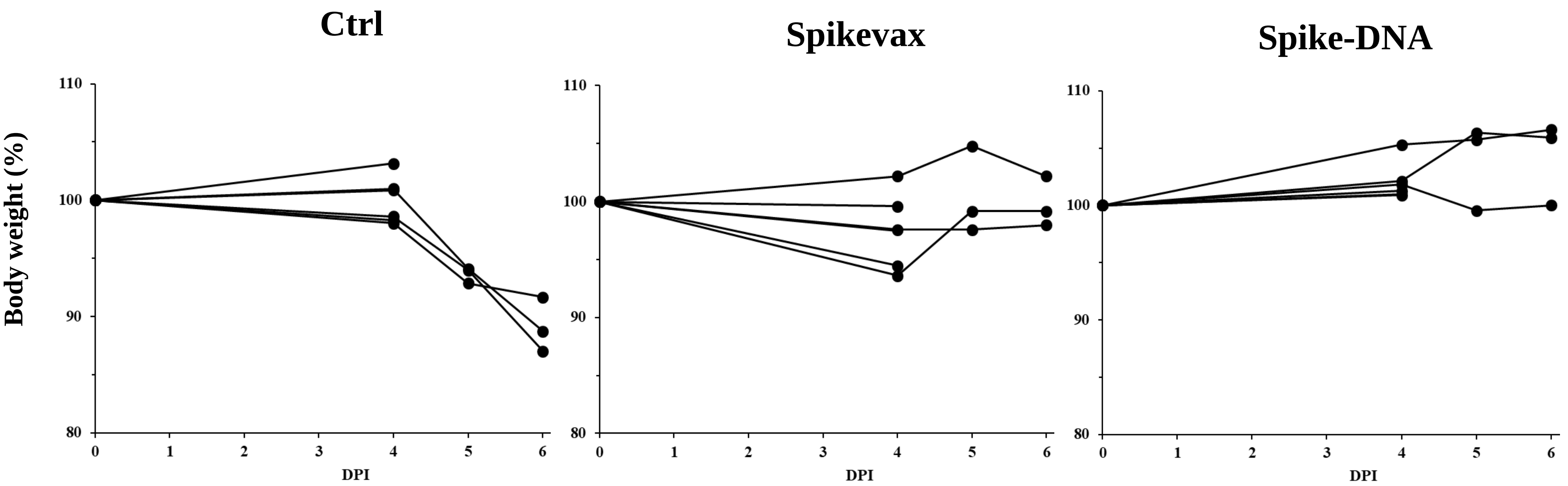

Supplementary Figure S3. Relative weight loss in mice infected by SARS-CoV-2 three months after vaccine booster. DPI: days post-infection.

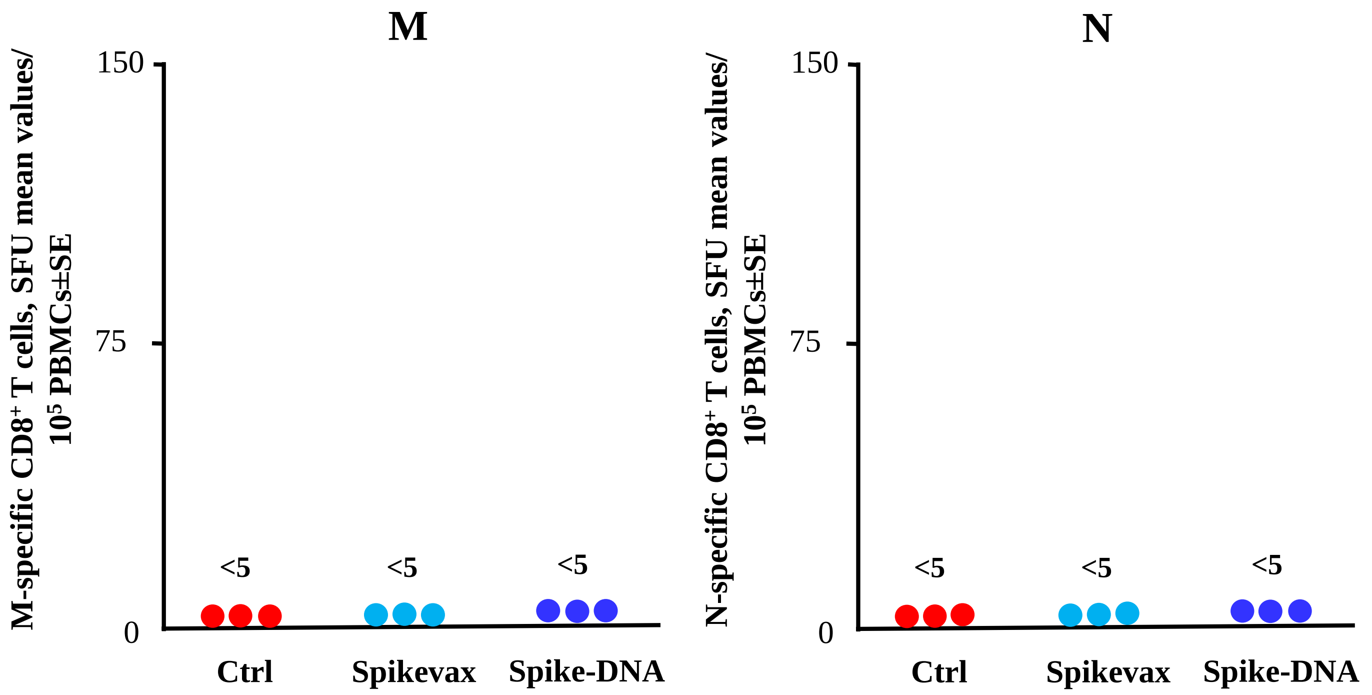

Supplementary Figure S4. Detection of both M-and N-specific circulatory CD8$^+$ T cells in K18-huACE2 mice three months after vaccine boosting and 4 days after infection. Shown are the results from IFN-γ ELISpot analysis carried out in 10^5 PBMCs incubated overnight with 5 μg/ml of either unrelated or M-specific peptide. Data were calculated as mean values of triplicates after subtraction of the mean SFUs detected in wells of PBMCs treated with an unspecific peptide (<3 for each cell culture tested).
